# Emission Spectroscopy as a Probe into Photoinduced Intramolecular Electron Transfer in Polyazine Bridged Ru(II),Rh(III) Supramolecular Complexes

**DOI:** 10.3390/ma3084328

**Published:** 2010-08-11

**Authors:** Travis A. White, Shamindri M. Arachchige, Baburam Sedai, Karen J. Brewer

**Affiliations:** Department of Chemistry, Virginia Polytechnic Institute and State University, Blacksburg, VA 24061-0212, USA

**Keywords:** emission spectroscopy, MLCT light absorbers, polyazine bridging ligands, supramolecular, intramolecular electron transfer

## Abstract

Steady-state and time-resolved emission spectroscopy are valuable tools to probe photochemical processes of metal-ligand, coordination complexes. Ru(II) polyazine light absorbers are efficient light harvesters absorbing in the UV and visible with emissive ^3^MLCT excited states known to undergo excited state energy and electron transfer. Changes in emission intensity, energy or band-shape, as well as excited state lifetime, provide insight into excited state dynamics. Photophysical processes such as intramolecular electron transfer between electron donor and electron acceptor sub-units may be investigated using these methods. This review investigates the use of steady-state and time-resolved emission spectroscopy to measure excited state intramolecular electron transfer in polyazine bridged Ru(II),Rh(III) supramolecular complexes. Intramolecular electron transfer in these systems provides for conversion of the emissive ^3^MLCT (metal-to-ligand charge transfer) excited state to a non-emissive, but potentially photoreactive, ^3^MMCT (metal-to-metal charge transfer) excited state. The details of the photophysics of Ru(II),Rh(III) and Ru(II),Rh(III),Ru(II) systems as probed by steady-state and time-resolved emission spectroscopy will be highlighted.

## 1. Introduction

Supramolecular complexes composed of multiple metal centers capable of light and/or redox induced processes are of interest in designing molecular machines [1]. In this sense, supramolecular complexes which couple multiple molecular components whose individual properties provide a unique function to the supramolecule are of wide interest [2]. Although the properties of the components may be perturbed upon coupling supramolecular assemblies, components bring to the molecular device a unique function typically retained by each subunit in the assembly. Supramolecular complexes that use light to initiate a function are coined photochemical molecular devices (PMDs). The appropriate assembly of molecular components within the PMDs can provide unique systems that perform complex tasks at the molecular level. Systems can be engineered to undergo vectoral electron transfer and migration of charge between appropriate electron donor (ED) and electron acceptor (EA) sites. This generation of charge separation and migration in molecular systems, induced by light absorption, is of considerable interest and applicable in many forums including artificial photosynthesis, molecular photovoltaics, solar energy conversion, and photodynamic therapy [2].

Emission spectroscopy provides an attractive tool to study the excited state charge transfer processes and interstate dynamics of supramolecules [3,4,5]. The photophysics and photochemistry of a variety of transition metal coordination complexes having metal-to-ligand charge-transfer (MLCT) transitions that are emissive in the solid state and/or solution at room temperature have been widely explored [1,2,4,6,7]. The coupling of these MLCT light absorbers to other units provides a means of deactivating the emissive ^3^MLCT excited states harvesting the stored energy which include intermolecular (*i.e.*, bimolecular deactivation) or intramolecular (*i.e.*, unimolecular decay) pathways. Reactions of the emissive MLCT excited states can lead to photoreactive species that mediate useful chemical reactions exploiting the long lived MLCT excited states of these chromophores. Understanding factors that control excited state deactivation processes allow the modulation of excited state properties and photoreactivity.

**Figure 1:**
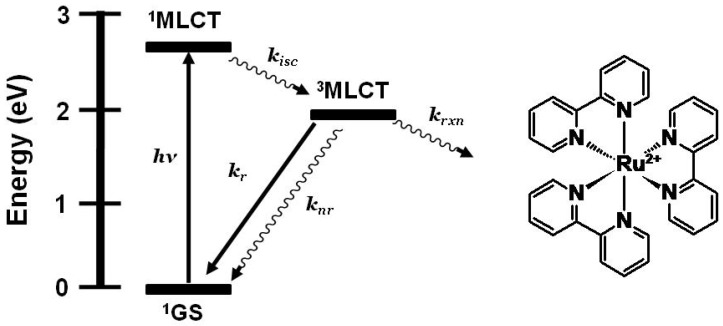
State diagram for [Ru(bpy)_3_]^2+^ (bpy = 2,2’-bipyridine). GS = ground state, MLCT = metal-to-ligand charge transfer excited state, *k_isc_* = intersystem crossing rate constant, *k_r_* = radiative decay rate constant, *k_nr_* = non-radiative decay rate constant, *k_rxn_* = photochemical reaction rate constant.

The prototypical light absorber [Ru(bpy)_3_]^2+^ (bpy = 2,2'-bipyridine) and related chromophores have been widely used as building blocks for synthesizing redox-active and luminescent supramolecular metal complexes. [Ru(bpy)_3_]^2+^ and related systems absorb light throughout the UV and visible, and typically populate the emissive ^3^MLCT excited state with unit efficiency providing ^3^MLCT emission at 605 nm with τ = 860 ns in CH_3_CN at room temperature [8]. Figure 1 shows the state diagram for [Ru(bpy)_3_]^2+^. Coupling multiple polyazine bridged Ru(II) centers gives systems that display redox-active and lumophoric properties [1,9,10]. While the study of supramolecular complexes bridged with polyazine ligands is an active field, the coupling of reactive metals is far less studied and provides a means to study intramolecular electron transfer processes for harvesting energy.

Our focus herein is on the use of emission spectroscopy to probe photoinduced intramolecular electron transfer of Ru(II) polyazine MLCT light absorbers coupled to electron accepting Rh(III) centers. A variety of Ru(II),Rh(III) and Ru(II),Rh(III),Ru(II) supramolecular complexes that possess ^3^MLCT emissions have been studied at room temperature and low temperature (usually 77 K) using steady-state and time-resolved emission spectroscopy to provide a probe into excited state dynamics of these systems. The pioneering work on intermolecular electron transfer between excited Ru(II) MLCT light absorbers and Rh(III) electron acceptors provides the framework for these studies [11,12,13,14].

### 1.1. Molecular Components of Ru(II),Rh(III) Supramolecular Complexes

The design of supramolecules for specific applications requires knowledge of the individual components and the role each subunit plays in the functioning of these supramolecular assemblies as well as the perturbations introduced upon coupling into the assembly [1,2,15]. The Ru(II),Rh(III) systems described herein undergo excitation to populate Ru-based MLCT excited states followed by intramolecular electron transfer to Rh to generate charge separation. Several factors can impact the properties of these systems: (1) the nature of the polyazine bridging ligand (BL) to connect molecular components, (2) the identity/ligand set of the Ru(II) light absorber, and (3) the Rh(III) electron acceptor (EA) identity/coordination environment. The coordination environment of each metal can modulate orbital energetics impacting the driving force for intramolecular electron transfer.

#### 1.1.1. Polyazine Bridging Ligands

**Figure 2:**
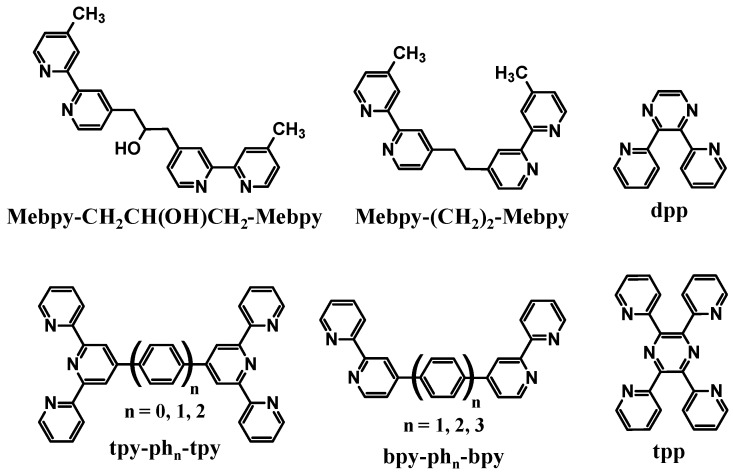
Polyazine bridging ligands.

**Figure 3:**
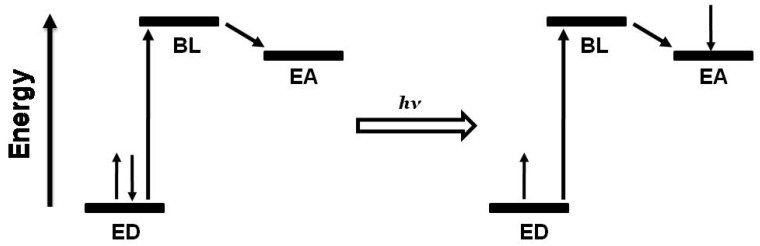
Schematic representation of ED-BL-EA orbital energetics showing excitation followed by intramolecular electron transfer. ED = electron donor, BL = bridging ligand, EA = electron acceptor.

Polyazine bridging ligands (BL) are commonly used to couple molecular components and are used herein to couple Ru LA to Rh EA subunits [1,16]. Polyazine BLs containing aliphatic or aromatic linkers are used in this forum, Figure 2. The BL typically forms coordinate covalent bonds to the Ru and Rh metal centers and therefore influences the properties of both of these subunits. BL π* orbitals are often the acceptor orbitals for the optically populated MLCT excited state, playing a direct role as an intermediate acceptor in the excited state dynamics of these supramolecules, Figure 3.

The complexation of a polyazine BL with metals such as Ru(II) and Rh(III) results in a stabilization of the π* acceptor orbitals, perturbing the electron accepting properties of the BL. The BL mediates intercomponent communication between the ED and EA. Metal-metal coupling in multimetallic assemblies can vary from strong to negligible depending on the nature of the BL and metals [1,16,17]. Extended aliphatic or aromatic bridges provide complexes with spectroscopic and redox properties that are closely approximated by the additive properties of the monometallic synthons. Shorter aromatic bridging ligands provide complexes where the properties of the synthons are significantly perturbed by the supramolecular assembly [1,16,17].

#### 1.1.2. Ru(II) Light Absorbers/Electron Donors

**Figure 4:**
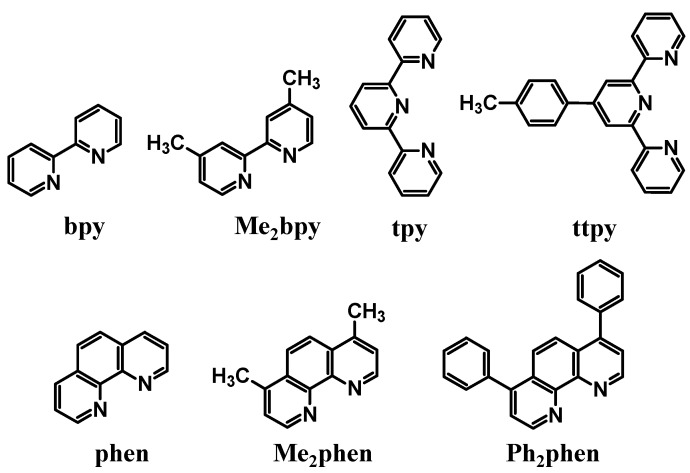
Polyazine terminal ligands.

Ru(II) light absorbers (LA) are often utilized to harness UV and visible energy and provide the emissive probe in mixed Ru(II),Rh(III) systems [1,5,18,19]. The prototypical LA, [Ru(bpy)_3_]^2+^, has properties that can be tuned by ligand variation. These metal-based LAs contain polyazine terminal ligands (TL), Figure 4, and BLs to satisfy the Ru coordination sphere and tune the energy of the MLCT excited states and redox properties. Photoexcitation of a Ru(II) LA populates π→π* (UV) or ^1^MLCT (visible) excited states that undergo intersystem crossing, with near unit efficiency, to populate the lowest lying, emissive ^3^MLCT excited states [20]. The BL incorporated in the Ru(II) LA moiety influences the energy of the lowest ^3^MLCT excited states which are typically BL(π*) based. The energy of the HOMO Ru(dπ) donor orbitals is tuned by the choice of TLs and BLs to allow the Ru(II) to function as an ED in the supramolecular complexes.

#### 1.1.3. Rh(III) Electron Acceptors

Rh(III) metal centers complexed to polyazine ligands function as EAs in these supramolecular assemblies, possessing low-lying, unoccupied Rh(dσ*) orbitals which affords directional flow of charge following optical excitation [17,21]. Bimolecular systems have been studied illustrating that excited state electron transfer from *[Ru(bpy)_3_]^2+^ to [Rh(bpy)_3_]^3+^ occurs leading to emission quenching of the Ru ^3^MLCT excited state [11,12,13,14]. This highlights the ability of Rh(III) to act as an EA unit to excited Ru LAs. Connecting the Rh(III) EA to the Ru(II) ED through the BL generates a donor-bridge-acceptor (ED-BL-EA) structural motif. Most systems employ the tris(bidentate) or bis(tridentate) coordination on Rh(III) typically preventing reactivity at the rhodium site, allowing simple intramolecular electron transfer [1].

### 1.2. Photoinitiated Electron Collection

**Figure 5:**
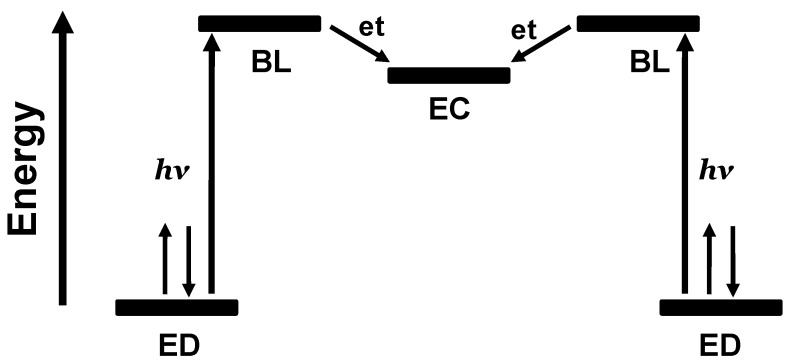
Schematic representation of the orbital energetics within a photoinitiated electron collector of the ED-BL-EC-BL-ED design. ED = electron donor, BL = bridging ligand, EC = electron collector, et = intramolecular electron transfer.

Photochemical molecular devices that collect reducing equivalents at a single site through photoactivated processes are photoinitiated electron collectors (PECs) [2]. Long term interest in this function results from the desire to use light energy to drive fuel producing multi-electron chemistry. The coupling of two molecular photovoltaics using a common EA that can collect multiple electrons produces a ED-BL-EC-BL-ED (where EC = electron collector) assembly capable of electron collection at the central EC sub-unit, Figure 5. Early PECs incorporated extended polyazine bridging ligands [22,23] or a BL-Ir^III^Cl_2_-BL moiety [24] as ECs. Changing the central metal from Ir(III) to Rh(III) allows electron collection on a metal center at the Rh site [25]. The Rh(III)-based PECs are shown to be active photocatalysts for the multielectron reduction of H_2_O to produced H_2_ [26,27,28,29].

### 1.3. Photoinduced Intramolecular Electron Transfer

Electronic excited states have significant added energy as a result of optical excitation and this energy can be harvested through electron or energy transfer and photoreactions. Electronic excited states are more powerful oxidizing and reducing agents due to the lower energy hole and high energy electron produced by photoexcitation. Photoexcitation throughout the UV and visible result in generation of the ^3^MLCT excited state of Ru(II) polyazine complexes typically with unit efficiency, equation 1.

[Ru(bpy)_3_]^2+^ + hν → *[Ru(bpy)_3_]^2+^ (1)

#### 1.3.1. Thermodynamics of Excited State Electron Transfer

The ^3^MLCT excited states of Ru(II) polyazine systems are known to undergo excited state oxidative quenching (equation 2) or reductive quenching (equation 3).

*[Ru^II^(bpy)_3_]^2+^ + EA → [Ru^III^(bpy)_3_]^3+^ + EA^−^ (2)

*[Ru^II^(bpy)_3_]^2+^ + ED → [Ru^II^(bpy)_2_(bpy^−^)]^1+^ + ED^+^ (3)

The thermodynamic driving force for oxidative (equation 4) and reductive (equation 5) quenching can be calculated using the ground state redox potentials and the E^0-0^ energy of the ^3^MLCT excited state (where LA = Ru(II) polyazine light absorber, E(LA^+^/LA) is the ground state oxidation potential and E(LA/LA^-^) is the ground state reduction potential).

E(*LA/LA^−^) ≈ E(LA^+^/LA) + E^0-0^ (4)

E(*LA/LA^+^) ≈ E(LA/LA^-^) − E^0-0^ (5)

The reactions of such monometallic Ru(II) polyazine complexes rely on diffusional contact to give rise to intermolecular electron transfer during the excited state lifetime of the LA. Coupling electron donors or acceptors are possible to produce supramolecular systems. The coupling of Ru(II) polyazine light absorbers to Rh(III) electron acceptors provides for LA-EA supramolecules. Here excitation occurs at the LA subunit to the produce the ^3^MLCT excited state of the LA, equation 6.

LA-EA + hν → *LA-EA
(6)

Excited state intramolecular electron transfer can occur to produce an oxidized Ru center and reduced Rh center, equation 7.

*LA-EA → LA^+^-EA^−^ (7)

The thermodynamic driving force for this process is given in equation 8 with the potentials being the ground state oxidation potential of the LA fragment and the reduction potential of the EA fragment and E_IP_ the Coulombic stabilization energy of the product [30].

ΔG° ≈ −E^0-0^ − E(EA/EA^−^) + E(LA^+^/LA) − E_IP_ (8)

#### 1.3.2. Factors Influencing the Rate of Electron Transfer

The rate constant for the electron transfer process (*k_et_*) can be related to this thermodynamic driving force (ΔG^◦^), the average nuclear frequency factor (ν_N_), the electronic transmission coefficient (κ) and the total reorganizational energy (λ), equation 9 [31,32].

(9) $$k_{et}=\nu_{N}\kappa e^{\frac{-\Delta G^{‡}}{RT}}\;\Delta G^{‡}=\left(\frac{\lambda }{4}\right){\left(1+\frac{\Delta G{}^{\circ}}{\lambda }\right)}^{2}$$

This provides for the bell-shaped relationship between ln *k* and ΔG^◦^ that provides for an increase in *k_et_* as driving force increases in the Marcus “normal” region and the decrease in *k_et_* with increasing driving force at large driving forces, giving rise to the Marcus “inverted” region. The total reorganizational energy is a sum of inner and outer sphere reorganizational energy with outer sphere being the dominate factor. This energy increases as the distance between the donor and acceptor increases. Consideration of electronic interaction between the donor and acceptor wavefunctions are needed to provide for a mechanism of electron transfer and in most systems it is reasonable to assume a small amount of such mixing occurs. Transferring an electron can occur directly from the donor to the acceptor (superexchange mechanism) or by sequential localization of the electron from the donor to the bridge to the acceptor (electron hopping mechanism) [1,17].

The molecular components utilized to construct the supramolecular assemblies modulate the rate of intramolecular electron transfer (*k_et_*). To promote excited state electron transfer, supramolecular design is used to facilitate coupling and thermodynamically favorable electron transfer from the ED to the EA. The BL mediates intercomponent communication between the ED and EA. Reports of factors controlling intramolecular electron transfer are available [1,16,17,32,33]. Using a weakly coupling description of molecular components, *k_et_* is proportional to the square of the electron donor-electron acceptor electronic coupling matrix element (H_DA_), equation 10 [1,32].

(10) $$k_{et}=\left(\frac{2\pi }{\text{ħ}}\right){H_{DA}}^{2}\left(\frac{1}{\sqrt{4\pi \lambda RT}}\right)e^{\left[\frac{-{\left(\Delta G{}^{\circ}+\;\lambda \right)}^{2}}{4\lambda RT}\right]}$$

In equation 10, H_DA_ is modulated the internuclear distance between ED and EA (r_DA_) and a term β that relates interactions of spacer units in extended bridging ligands, λ is the total reorganization energy, ΔG° Gibbs free energy of electron transfer between ED and EA. This illustrates that the nature of the bridging ligand between the Ru polyazine LA and the Rh EA unit will impact the rate of intramolecular electron transfer such that enhanced electronic coupling of the donor and acceptor orbitals will provide for an increase in the rate of electron transfer and reduced donor-acceptor distance will facilitate electron transfer.

#### 1.3.3. Emission Spectroscopy as a Probe of Electron Transfer

**Figure 6:**
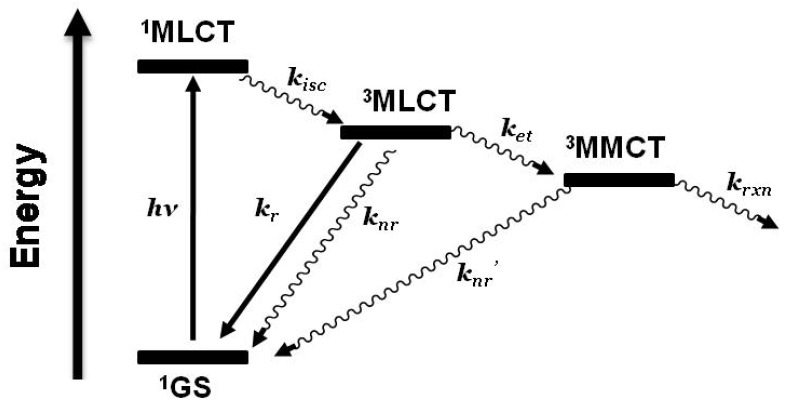
State diagram for Ru(II),Rh(III) supramolecular assemblies. GS = ground state, MLCT = metal-to-ligand charge transfer excited state, MMCT = metal-to-metal charge transfer excited state, *k_isc_* = intersystem crossing rate constant, *k_r_* = radiative decay rate constant, *k_nr_* = non-radiative decay rate constant, *k_et_* = intramolecular electron transfer rate constant, *k_rxn_* = photochemical reaction rate constant.

Emission spectroscopy is often used to probe intramolecular electron transfer within a supramolecular assembly possessing Ru(II) MLCT light absorbers [3,4]. The excited state properties of the individual molecular components are used as a model to compare with the multi-component assembly. In the Ru(II),Rh(III) and Ru(II),Rh(III),Ru(II) complexes discussed herein, the emissive excited state is ^3^MLCT in nature and model systems can be constructed. Careful analysis of the model systems and associated assumptions is critical to using emission spectroscopy as a probe of intramolecular electron transfer. The energy and nature of the emissive state of the model must closely match that of the supramolecular assembly for accurate determination of the rate of electron transfer. Ru(II),Rh(III) supramolecular assemblies with orbital energetics appropriate for thermodynamically favorable intramolecular electron transfer provide for systems with low-lying MLCT and MMCT excited states, Figure 6.

Steady-state emission spectroscopy shows a substantial decrease in the quantum yield of emission ($\Phi^{\mathrm{em}})$ in the supramolecular assembly compared with the respective model complexes ($\Phi_{0}^{\mathrm{em}}$) at room temperature when intramolecular electron transfer occurs, equations 11 and 12.

(11) $$\Phi^{\mathrm{em}}=\frac{k_{r}}{k_{r}+k_{nr}+k_{et}}$$

(12) $$\Phi_{0}^{em}=\frac{k_{r}}{k_{r}+k_{nr}}$$

Using a ratio of the inverse of the two quantum yields allows the determination of *k_et_*. This calculation assumes a good model where the rate of radiative (*k_r_*) and non-radiative (*k_nr_*) deactivation of the ^3^MLCT excited state does not vary between the supramolecular assembly and the model system. Time-resolved emission spectroscopy can also be used to calculate the rate of intramolecular electron transfer, k_et_, as shown in equations 13-15,

(13) $$\tau =\frac{1}{k_{r}+k_{nr}+k_{et}}$$

(14) $$\tau_{0}=\frac{1}{k_{r}+k_{nr}}$$

(15) $$k_{et}=\;\frac{1}{\tau }-\;\frac{1}{\tau_{0}}$$

where *τ* and *τ_0_* are the measured ^3^MLCT excited state lifetimes of the supramolecular assembly and the appropriate model, respectively. The room temperature values for *τ* are substantially smaller than *τ_0_* in these Ru(II),Rh(III) supramolecules, supporting quenching of the ^3^MLCT emissive excited state via intramolecular electron transfer. When the time-resolved emission decay in a rigid matrix at 77 K of the supramolecular assembly and the model are the same this verifies that intramolecular electron transfer to populate the non-emissive ^3^MMCT excited state occurs at room temperature and is impeded at 77 K. Electron transfer is impeded at 77 K in a rigid media while energy transfer is not, therefore allowing the determination of the quenching mechanism at room temperature.

## 2. Ru(II),Rh(III) Bimetallic Complexes

The Ru(II),Rh(III) bimetallic motif couples a Ru(II) ED to a Rh(III) EA by a polyazine BL. Population of the Ru(II)-based ^3^MLCT excited states can be followed by intramolecular electron transfer to the unoccupied Rh(dσ*) electron acceptor orbitals. In the systems discussed, the BLs contain aliphatic-linkers (methylene groups), aromatic-linkers (phenylene groups) or a pyrazine unit to connect the ED and EA units (see Figure 2 for structures of BLs). The choice of BL within the supramolecular assembly strongly influences the degree of electronic communication between the Ru(II) and Rh(III) metal centers typically serves as the intermediate acceptor in the emissive ^3^MLCT excited state and changes the rate of intramolecular electron transfer. Appendix 1 contains a summary of the reported photophysical data for the Ru(II),Rh(III) bimetallic complexes and respective model systems discussed.

### 2.1. Polyazine Bridging Ligands Containing Aliphatic Linkers

**Figure 7:**
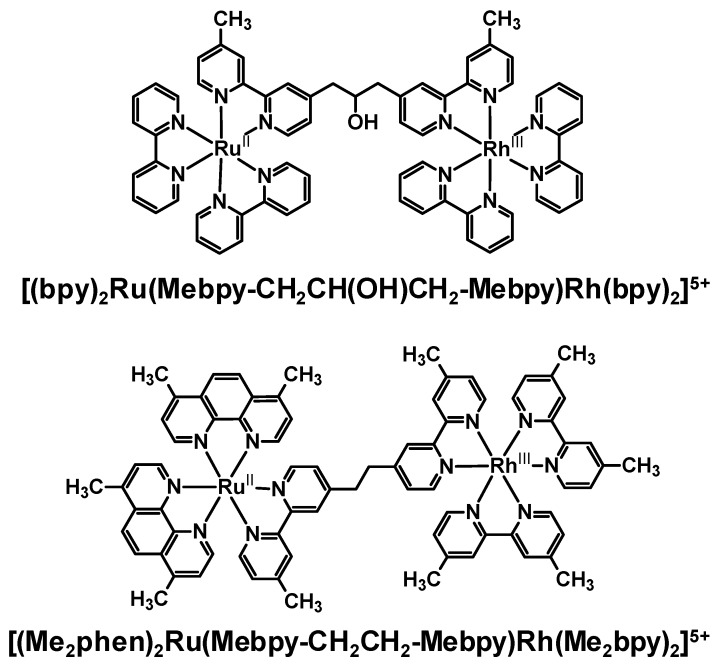
Ru(II),Rh(III) bimetallic complexes containing an aliphatic-linked BL.

A few early studies used steady-state and time-resolved emission spectroscopy to probe the intramolecular electron transfer within Ru(II),Rh(III) bimetallic complexes coupled by polyazine BLs with aliphatic linkers. The Ru(II),Rh(III) complexes, [(bpy)_2_Ru(Mebpy-CH_2_CH(OH)CH_2_-Mebpy)Rh(TL)_2_]^5+^ (TL = bpy, or phen) [34] and [(Me_2_phen)_2_Ru(Mebpy-CH_2_CH_2_-Mebpy)Rh(Me_2_bpy)_2_]^5+^, [35] Figure 7, are of the ED-BL-EA structural motif. Polyazine BLs containing aliphatic linkers results in weakly coupled Ru(II) and Rh(III) molecular components. The monometallic complexes, [(bpy)_2_Ru(Me_2_bpy)]^2+^ and [(Me_2_phen)_2_Ru(Mebpy-CH_2_-CH_2_-Mebpy)]^2+^, were used as model systems to evaluate *k_et_* for the analogous Ru(II),Rh(III) complexes due to their similar nature and energy of the emissive states. It should be considered that the lack of metal complexation to the remote site of the BL may influence the observed properties of these systems.

In both [(bpy)_2_Ru(Mebpy-CH_2_CH(OH)CH_2_-Mebpy)Rh(TL)_2_]^5+^ and [(Me_2_phen)_2_Ru(Mebpy-CH_2_CH_2_-Mebpy)Rh(Me_2_bpy)_2_]^5+^, the electrochemical and spectroscopic properties are not perturbed relative to their respective monometallic models due to weak coupling through the bridge supporting the use of Ru monometallic model systems. The [(bpy)_2_Ru(Mebpy-CH_2_CH(OH)CH_2_-Mebpy)Rh(TL)_2_]^5+^ systems where TL = bpy or phen, emit at 610 nm with the emission intensity in water at room temperature decreased by 88 % and 83 %, respectively, compared to [(bpy)_2_Ru(Me_2_bpy)]^2+^. This is concluded to result from intramolecular electron transfer quenching of the ^3^MLCT excited state to generate the ^3^MMCT state [34]. The rate of intramolecular electron transfer from the excited Ru(II) component to the electron accepting Rh(III) components are 1.4 × 10^7^ s^−1^ (TL = bpy) and 1.1 × 10^7^ s^−1^ (TL = phen). Similarly the [(Me_2_phen)_2_Ru(Mebpy-CH_2_CH_2_-Mebpy)Rh(Me_2_bpy)_2_]^5+^ complex displays efficient intramolecular quenching of the Ru(II)-based MLCT excited state by electron transfer to the Rh(III) component. In room temperature CH_3_CN, the [(Me_2_phen)_2_Ru(Mebpy-CH_2_CH_2_-Mebpy)Rh(Me_2_bpy)_2_]^5+^ supramolecule ($\lambda_{3_{MLCT}}^{em}$ = 610 nm, $\Phi_{3_{MLCT}}^{em}$ = 7.6 × ^10−4^, τ = 6 ns) exhibits ca. 99% emission quenching compared to the [(Me_2_phen)_2_Ru(Mebpy-CH_2_CH_2_-Mebpy)]^2+^ model complex ($\lambda_{3_{MLCT}}^{em}$ = 610 nm, $\Phi_{3_{MLCT}}^{em}$ = 0.11, τ = 1.8 μs) and yields a *k_et_* value of 1.7 × 10^8^ s^−1^ [35]. The 77 K steady-state and time-resolved emission spectra are nearly identical for the supramolecular assembly [(Me_2_phen)_2_Ru(Mebpy-CH_2_CH_2_-Mebpy)Rh(Me_2_bpy)_2_]^5+^ ($\lambda_{3_{MLCT}}^{em}$ = 575 nm; τ = 6.8 μs) and the model [(Me_2_phen)_2_Ru(Mebpy-CH_2_CH_2_-Mebpy)]^2+^ ($\lambda_{3_{MLCT}}^{em}$ = 575 nm; τ = 7 μs) in a 4:1 EtOH/MeOH as a result of inhibition of intramolecular electron transfer in a low temperature rigid matrix.

**Figure 8:**
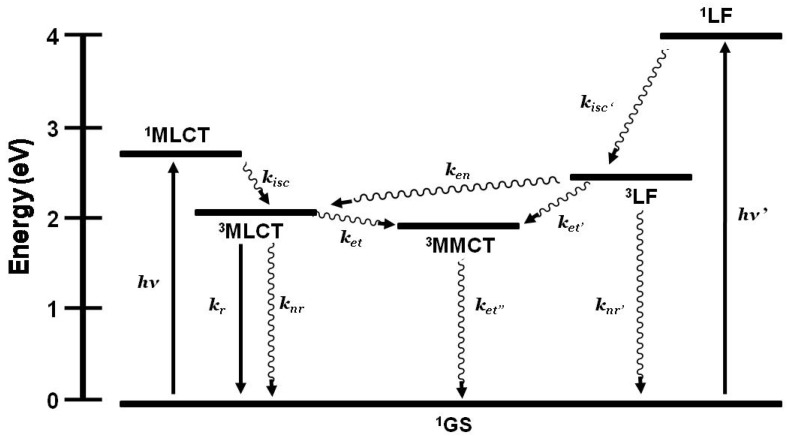
State diagram of [(Me_2_phen)_2_Ru(Mebpy-CH_2_CH_2_-Mebpy)Rh(Me_2_bpy)_2_]^5+^ displaying possible excited state deactivating pathways. *k_isc_* = intersystem crossing rate constant, *k_r_* = radiative decay rate constant, *k_nr_* = non-radiative decay rate constant, *k_et_* = electron transfer rate constant, *k_en_* = energy transfer rate constant. Adapted from reference 35.

The study of the bimetallic complex [(Me_2_phen)_2_Ru(Mebpy-CH_2_CH_2_-Mebpy)Rh(Me_2_bpy)_2_]^5+^ considered the possibility of multiple room temperature intramolecular deactivation pathways and excitations, Figure 8 [35]. Selective photoexcitation of the Ru(II) or Rh(III) molecular components populates localized excited states. In this system the ^3^MLCT excited state lies above the ^3^MMCT state but below the ^3^LF state of the Rh(III). Electron transfer from the ^3^MLCT is thermodynamically favorable by −0.10 eV, slightly exergonic lying in the Marcus “normal” region [32]. Evidence of energy and electron transfer from the ^3^LF to the ^3^MLCT and ^3^MMCT excited states, respectively, was observed using transient absorption spectroscopy. In a rigid matrix, electron transfer deactivation pathways are inhibited and energy transfer is observed.

### 2.2. Polyazine Bridging Ligands Containing Aromatic Linkers

**Figure 9:**
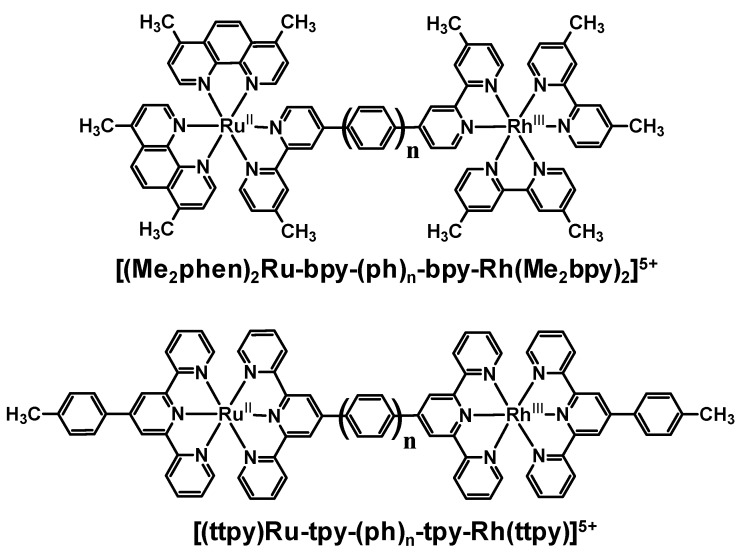
Ru(II),Rh(III) bimetallic complexes containing a phenylene-linked polyazine BL.

Ru(II)- and Rh(III)-polyazine components with tris-bidentate or bis-tridentate chelating schemes have been used to study the impact of phenylene-linkers on the steady-state and time-resolved emission properties of the supramolecular assemblies. Donor-acceptor distance (r_DA_) and attenuation (β) factors influence the rate of electron transfer in the series of Ru(II),Rh(III) bimetallic complexes [(Me_2_phen)_2_Ru-bpy-(ph)_n_-bpy-Rh(Me_2_bpy)_2_]^5+ ^and [(ttpy)Ru-tpy-(ph)_n_-tpy-Rh(ttpy)]^5+^, Figure 9 [36,37,38]. Intramolecular electron transfer was observed in the Ru(II),Rh(III) systems through quenching of the Ru based ^3^MLCT emission. The lack of a sufficient model for [(ttpy)Ru-tpy-tpy-Rh(ttpy)]^5+^ has resulted in limited conclusions for this complex [38].

The bis-tridentate supramolecular assemblies [(ttpy)Ru-tpy-(ph)_n_-tpy-Rh(ttpy)]^5+^ (where n = 0, 1, 2) were studied to probe the rate of intramolecular electron transfer as a function of the number of phenyl spacers [37,38]. These systems utilize the very short lived bis(tridentate) Ru chromophores. The monometallic, [Ru(ttpy)_2_]^2+^ was used to study emission spectroscopy for n = 1 or 2 bimetallics. Inclusion of the phenylene linker between the tpy-based BL decreases the electronic coupling of the Ru(II) and Rh(III) metal centers relative to the directly linked system, supported by the additive nature of the absorption spectra of the components in the supramolecular assembly. Emission from these bimetallics at room temperature, 150 K and 77 K are similar to the [Ru(ttpy)_2_]^2+^ model ($\lambda_{77K}$ = 629 nm and $\lambda_{150K}$ = 645 nm). At 150 K and 77 K, [(ttpy)Ru-tpy-(ph)-tpy-Rh(ttpy)]^5+^ and [(ttpy)Ru-tpy-(ph)_2_-tpy-Rh(ttpy)]^5+^ have similar values of *τ* to [Ru(ttpy)_2_]^2+^ ($\tau_{77K}$ = 13.5 μs and $\tau_{150K}$ = 3.2 μs). Room temperature excited state lifetime measurements in acetonitrile vary with the number of phenylene spacers (*τ* = 240 ps, [(ttpy)Ru-tpy-(ph)-tpy-Rh(ttpy)]^5+^; *τ* = 1.9 ns, [(ttpy)Ru-tpy-(ph)_2_-tpy-Rh(ttpy)]^5+^; *τ* = 860 ps, [Ru(ttpy)_2_]^2+^). The variation of the donor-acceptor distance in these systems changes the rate of electron transfer. The value of *k_et_* calculated for [(ttpy)Ru-tpy-(ph)-tpy-Rh(ttpy)]^5+^ was ≥ 3 × 10^9^ s^−1^ for n = 1 and < 5 × 10^8^ s^−1 ^for n = 2. Efficient intramolecular electron transfer quenching of the emissive Ru(II) ^3^MLCT excited state is observed in these systems.

[(ttpy)Ru-tpy-tpy-Rh(ttpy)]^5+^ displays intercomponent coupling as the electronic absorption spectrum displays a distinct red-shift of the lowest energy Ru→BL charge transfer transition band, similar to that observed for the analogous Ru(II),Ru(II) bimetallic, [(ttpy)Ru-tpy-tpy-Ru(ttpy)]^4+^ [39]. In a 150 K fluid solution and 77 K rigid matrix of 4:1 EtOH/MeOH, the energy of emission for [(ttpy)Ru-tpy-tpy-Rh(ttpy)]^5+^ and [(ttpy)Ru-tpy-tpy-Ru(ttpy)]^4+^ are similar ($\lambda_{150K}$ = 708 nm and $\lambda_{77K}$ = 674 nm). The excited state lifetimes at 77 K are similar as well (τ = 12.5 and 12.9 μs, respectively). Increasing the temperature to 150 K displays a substantial decrease in the [(ttpy)Ru-tpy-tpy-Rh(ttpy)]^5+^ lifetime (τ < 0.1 μs) compared to the Ru(II),Ru(II) bimetallic (τ = 3.5 μs). At room temperature, the use of the Ru(II),Ru(II) bimetallic as a model complex for [(ttpy)Ru-tpy-tpy-Rh(ttpy)]^5+^ fails due to a substantial red-shift in emission energy and decrease in excited state lifetime for [(ttpy)Ru-tpy-tpy-Rh(ttpy)]^5+^ in comparison to the Ru(II),Ru(II) bimetallic.

The tris-bidentate complexes [(Me_2_phen)_2_Ru-bpy-(ph)_n_-bpy-Rh(Me_2_bpy)_2_]^5+^ (where n = 1, 2, or 3) display a distance dependence on the rate of intramolecular electron transfer probed by emission spectroscopy [36]. The electronic coupling between the Ru(II) and Rh(III) molecular components is sufficiently weak that the corresponding monometallic analogues [(Me_2_phen)_2_Ru-bpy-(ph)_n_-bpy]^2+^ are used as model systems. The emission energy for the [(Me_2_phen)_2_Ru-bpy-(ph)_n_-bpy-Rh(Me_2_bpy)_2_]^5+^ complexes was found to be similar ($\lambda_{3_{MLCT}}^{em}$ = 640-652 nm at room temperature; $\lambda_{3_{MLCT}}^{em}$ = 600-615 nm at 77 K) and close to that of the Ru(II) monometallic synthons used as models ($\lambda_{3_{MLCT}}^{em}$ = 642-652 nm at room temperature; $\lambda_{3_{MLCT}}^{em}$ = 600-610 nm at 77 K). The time-resolved emission studies of the Ru(II),Rh(III) supramolecules at room temperature show a lifetime dependence on the BL length or donor-acceptor distance; *τ* = 0.360 ns, 2.3 ns, and 94 ns for n = 1, 2, or 3, respectively. The corresponding *k_et_* for the Ru(II),Rh(III) complexes decreases by ca. an order of magnitude with the addition of each phenylene spacer, *k_et_* = 3.0 × 10^9^ s^−1^, 4.3 × 10^8^ s^−1^ and 1.0 × 10^7^ s^−1^, respectively. Comparing the analogous aliphatic bridged Ru(II),Rh(III) bimetallic complex [(Me_2_phen)_2_Ru(Mebpy-CH_2_CH_2_-Mebpy)Rh(Me_2_bpy)_2_]^5+^ suggests that the phenylene-containing BL facilitates intramolecular electron transfer from the Ru(II) MLCT LA to the Rh(III) electron acceptor. This may result from localization of the excited electron in the ^3^MLCT state on the phenylene units decreasing the distance between the formal donor and acceptor in this phenylene bridged motif.

### 2.3. Polyazine Bridging Ligands Containing Pyrazine Linkers

**Figure 10:**
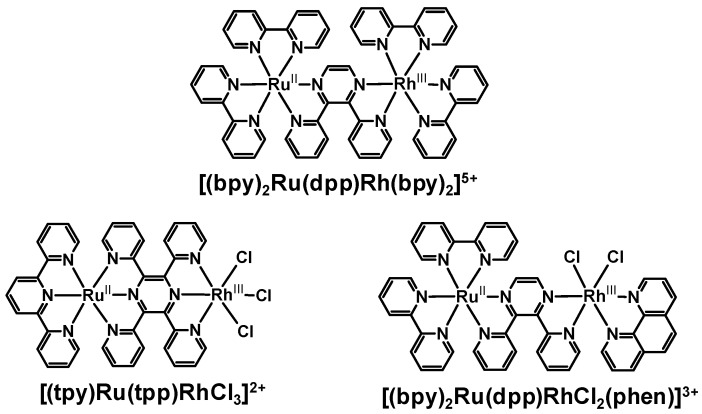
Ru(II),Rh(III) bimetallic complexes containing a pyrazine-linked BL.

Ru(II),Rh(III) complexes bridged by dpp (2,3-bis(2-pyridyl)pyrazine) display electronic communication between the coupled metal centers and show perturbed electrochemical and spectroscopic properties relative to the Ru(II) or Rh(III) monometallic subunits, Figure 10 [40,41,42]. Coupling two electropositive metals through a dpp bridge results in significant stabilization of the dpp(π*) orbital [1,9]. This is manifested by a shift in the ^1^MLCT absorption and ^3^MLCT emission energy. These strongly coupled systems require careful consideration of the model system. The energy of the emissive ^3^MLCT excited states in Ru(II),Rh(III) supramolecules is quite similar to that observed in the related Ru(II),Ru(II) bimetallic systems which lack the low lying ^3^MMCT excited state and do not undergo intramolecular electron transfer. The corresponding Ru(II),Ru(II) complexes [(TL)_2_Ru(BL)Ru(TL)_2_]^4+^ are used as models to calculate the rate of intramolecular electron transfer in strongly coupled systems. The stabilization of the emissive ^3^MLCT excited states in these systems moves it closer to the energy of the ^3^MMCT state generated by intramolecular electron transfer reducing the driving force for electron transfer.

**Figure 11:**
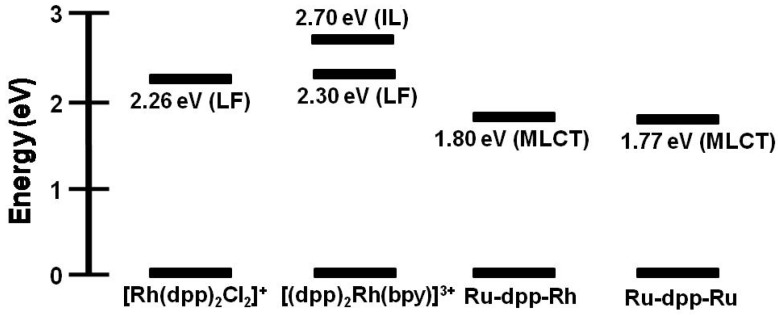
Relative E^0-0^ energies of excited states associated with mono- and bimetallic species. Values of E^0-0^ taken from 77 K emission measurements in 4:1 EtOH/MeOH glass. dpp = 2,3-bis(2-pyridyl)pyrazine, IL = intraligand excited state, LF = ligand field excited state, MLCT = metal-to-ligand charge transfer excited state. Adapted from [40].

The [(bpy)_2_Ru(dpp)Rh(bpy)_2_]^5+^ complex displays emission quenching of the ^3^MLCT excited state through intramolecular electron transfer [40]. The photophysical properties of both the Ru and Rh monometallic synthons and Ru(II),Ru(II) bimetallic complex were compared with those of the Ru(II),Rh(III) supramolecular assembly. Monometallic Rh(III)-containing complexes [Rh(dpp)_2_Cl_2_]^+^ and [(dpp)Rh(bpy)_2_]^3+^ emit from ligand field (^3^LF) and intraligand (^3^IL) excited states at room temperature, respectively. At 77 K, [Rh(dpp)_2_Cl_2_]^+^ emits from the same ^3^LF excited states while [(dpp)Rh(bpy)_2_]^3+^ is reported to show a strong ^3^IL emission and a weak ^3^LF emission at lower energy [3,43]. The Ru(II)-containing complexes [(bpy)_2_Ru(dpp)]^2+^ and [(bpy)_2_Ru(dpp)Ru(bpy)_2_]^4+^ exhibit emissions that are Ru→dpp ^3^MLCT in nature at room temperature and 77 K, typical of Ru(II)-polyazine chromophores. A state diagram correlating the energies of the various mono- and bimetallic complexes is shown in Figure 11. Coordination of a (bpy)_2_Ru^II^(dpp) moiety to Rh^III^(bpy)_2_ to generate [(bpy)_2_Ru(dpp)Rh(bpy)_2_]^5+^ ($\lambda_{3_{MLCT}}^{em}$ = 778 nm; *τ* = 37 ns) provides a supramolecular complex with an emissive ^3^MLCT excited state similar in energy to the [(bpy)_2_Ru(dpp)Ru(bpy)_2_]^4+^ analogue ($\lambda_{3_{MLCT}}^{em}$ = 790 nm; *τ_0_* = 140 ns). In room temperature CH_3_CN, the ^3^MLCT emission of [(bpy)_2_Ru(dpp)Rh(bpy)_2_]^5+^ is strongly quenched relative to [(bpy)_2_Ru(dpp)Ru(bpy)_2_]^4+^, attributed to an intramolecular electron transfer. This emission quenching provides a rate for electron transfer from the ^3^MLCT excited state to generate the ^3^MMCT state of *k_et_* = 2.83 × 10^7^ s^−1^. This indicates efficient intramolecular electron transfer in this strongly coupled system.

## 3. Ru(II),Rh(III),Ru(II) Trimetallic Complexes

**Figure 12:**
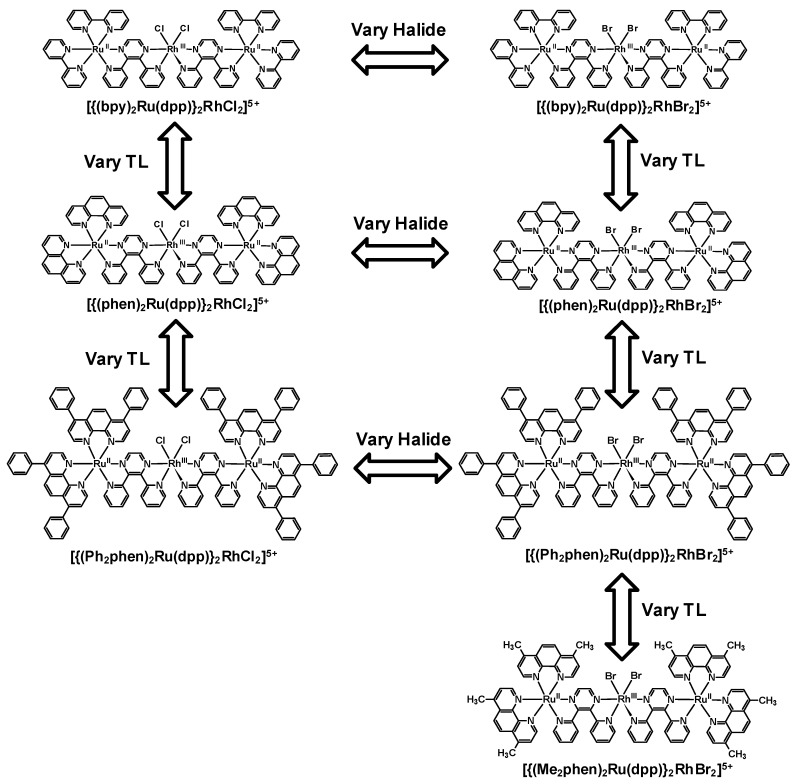
Polyazine-bridged Ru(II),Rh(III),Ru(II) supramolecular complexes with varying components. TL = terminal ligand.

**Figure 13:**
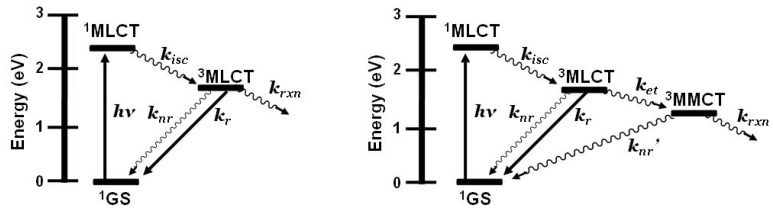
State diagram of Ru(II),Ru(II) bimetallic (left) and Ru(II),Rh(III),Ru(II) trimetallic (right). GS = ground state, MLCT = metal-to-ligand charge transfer excited state, MMCT = metal-to-metal charge transfer excited state, *k_isc_* = intersystem crossing rate constant, *k_r_* = radiative decay rate constant, *k_nr_* = non-radiative decay rate constant, *k_et_* = intramolecular electron transfer rate constant, *k_rxn_* = photochemical reaction rate constant.

Structurally diverse Ru(II),Rh(III),Ru(II) PECs [{(TL)_2_Ru(dpp)}_2_RhX_2_]^5+^ (X = halide, TL = bpy, phen, Ph_2_phen, Me_2_phen) that incorporate two Ru(II) LAs covalently bound to a Rh(III) EC through polyazine BLs have been reported, Figure 12 [25,26,27,29]. The trimetallic supramolecules contain Ru(II) and Rh(III) molecular components that display electrochemical and spectroscopic perturbations relative to their respective monometallic analogues. The Ru(II),Ru(II) systems that lack an EC unit are used as models for photophysical studies given the similar nature and energy of the emissive Ru(dπ)→dpp(π*) excited states. Electrochemical analysis of these dpp-bridged trimetallic complexes displays Ru(II) HOMOs and Rh(III) LUMOs suggesting the population of low-lying Ru(dπ)→Rh(dσ*) ^3^MMCT excited states is thermodynamically favorable from the optically populated ^3^MLCT excited states. Photoexcitation of the Ru(II) LA unit populates emissive Ru(dπ)→dpp(π*) ^3^MLCT excited states. A reduction in $\Phi^{em}$ and τ relative to the corresponding Ru(II),Ru(II) model system is observed, indicative of intramolecular electron transfer from the ^3^MLCT excited state to populate the energetically close Ru(dπ)→Rh(dσ*) ^3^MMCT excited state. Variation of the photophysical properties with component modification at room temperature and 77 K is reported. State diagrams for [{(TL)_2_Ru(dpp)}_2_RhX_2_]^5+^ Ru(II),Rh(III),Ru(II) supramolecules and [(TL)_2_Ru(dpp)Ru(TL)_2_]^4+^ model Ru(II),Ru(II) complexes are shown in Figure 13. Appendix 1 summarizes the photophysical properties of Ru(II),Rh(III),Ru(II) and Ru(II),Ru(II) model systems at room temperature and 77 K.

**Figure 14:**
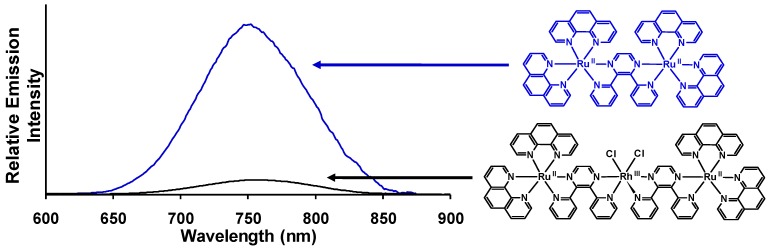
Emission spectra of the trimetallic complex [{(phen)_2_Ru(dpp)}_2_RhCl_2_]^5+^ (**―**) and the corresponding [(phen)_2_Ru(dpp)Ru(phen)_2_]^4+^ model (**―**) at room temperature in acetonitrile (phen = 1,10-phenanthroline, dpp = 2,3-bis(2-pyridyl)pyrazine). Emission spectra are corrected for PMT response.

Room temperature and 77 K emission was used to probe intramolecular electron transfer to populate the ^3^MMCT excited states within [{(bpy)_2_Ru(dpp)}_2_RhCl_2_]^5+^. The room temperature emission spectrum of [{(bpy)_2_Ru(dpp)}_2_RhCl_2_]^5+^ ($\lambda_{3_{MLCT}}^{em}$ = 776 nm; $\Phi_{3_{MLCT}}^{em}$ = 2.6 × 10^−4^) displays 73 % quenching of the emissive ^3^MLCT excited state relative to the model [(bpy)_2_Ru(dpp)Ru(bpy)_2_]^4+^ ($\lambda_{3_{MLCT}}^{em}$ = 752 nm; $\Phi_{3_{MLCT}}^{em}$ = 9.8 × 10^−4^) [29]. A concurrent reduction in the excited state lifetime of [{(bpy)_2_Ru(dpp)}_2_RhCl_2_]^5+^ (τ = 38 ns) is observed with respect to [(bpy)_2_Ru(dpp)Ru(bpy)_2_]^4+^ (τ = 140 ns) in acetonitrile. This data supports intramolecular electron transfer to populate the ^3^MMCT state with *k_et_* = 1.9 × 10^7^ s^−1^. The Ru(dπ)→dpp(π*) ^3^CT excited state shifts to higher energy at 77 K in a 4:1 EtOH/MeOH rigid matrix ($\lambda_{3_{MLCT}}^{em}$ = 730 nm for [{(bpy)_2_Ru(dpp)}_2_RhCl_2_]^5+^ and $\lambda_{3_{MLCT}}^{em}$ = 696 nm for [(bpy)_2_Ru(dpp)Ru(bpy)_2_]^4+^) with similar excited state lifetimes, 1.9 μs and 2.4 μs, respectively [42]. Changing the TL from bpy to phen in this structural motif results in [{(phen)_2_Ru(dpp)}_2_RhCl_2_]^5+^ ($\lambda_{3_{MLCT}}^{em}$ = 760 nm; $\Phi_{3_{MLCT}}^{em}$ = 2.2 × 10^−4^) which displays 86% quenching of the ^3^MLCT excited state relative to the model system [(phen)_2_Ru(dpp)Ru(phen)_2_]^4+^ ($\lambda_{3_{MLCT}}^{em}$ = 750 nm; $\Phi_{3_{MLCT}}^{em}$ = 1.6 × 10^−3^) at room temperature in CH_3_CN, Figure 14 [29]. Reduction of the excited state lifetimes in room temperature CH_3_CN for [{(phen)_2_Ru(dpp)}_2_RhCl_2_]^5+^ (τ = 35 ns) with respect to [(phen)_2_Ru(dpp)Ru(phen)_2_]^4+^ ( τ = 170 ns) is observed and provides *k_et_* = 2.3 × 10^7^ s^−1^. The 77 K emission of [{(phen)_2_Ru(dpp)}_2_RhCl_2_]^5+^ ($\lambda_{3_{MLCT}}^{em}$ = 706 nm; τ = 1.8 μs) is similar in energy and excited state lifetime to the model [(phen)_2_Ru(dpp)Ru(phen)_2_]^4+^ bimetallic complex ($\lambda_{3_{MLCT}}^{em}$ = 695 nm; τ = 2.0 μs) with electron transfer prohibited in a rigid matrix. Similar *k_et_* values are seen in these TL = bpy or phen Ru(II),Rh(III),Ru(II) systems with dpp bridges as in the Ru(II),Rh(III) dpp and tpp bridged systems discussed above (*k_et_* ≈ 10^7^ s^−1^).

The use of the Ph_2_phen TL in the Ru(II),Rh(III),Ru(II) supramolecular assemblies imparts somewhat surprising excited state properties despite the emissive state being formally Ru(dπ)→dpp(π*) CT in nature [44]. TL variation can have a substantial impact on excited state properties resulting from a significant TL contribution to the formally Ru(dπ) HOMO donor orbitals in this structural motif. In room temperature CH_3_CN, emission of [{(Ph_2_phen)_2_Ru(dpp)}_2_RhCl_2_]^5+^ is $\lambda_{3_{MLCT}}^{em}$ = 770 nm with $\Phi_{3_{MLCT}}^{em}$ = 2.4 × 10^−4^. Comparing the Ru(II),Rh(III),Ru(II) supramolecule to the model system [(Ph_2_phen)_2_Ru(dpp)Ru(Ph_2_phen)_2_]^4+^ ($\lambda_{3_{MLCT}}^{em}$ = 754 nm and $\Phi_{3_{MLCT}}^{em}$ = 1.7 × 10^−3^) shows that [{(Ph_2_phen)_2_Ru(dpp)}_2_RhCl_2_]^5+^ displays a ^3^MLCT emission with $\Phi_{3_{MLCT}}^{em}$ that is quenched 86 % relative to the model Ru(II),Ru(II) system. The room temperature time-resolved emission lifetime is shortened for [{(Ph_2_phen)_2_Ru(dpp)}_2_RhCl_2_]^5+^ relative to [(Ph_2_phen)_2_Ru(dpp)Ru(Ph_2_phen)_2_]^4+^ (τ = 52 ns *vs.* 192 ns, respectively), which gives *k_et_* = 1.4 × 10^7^ s^−1^. Substituent variation to TL = Me_2_phen also displays changes in the excited state properties. [{(Me_2_phen)_2_Ru(dpp)}_2_RhBr_2_]^5+^ emits from Ru(dπ)→dpp(π*) excited states at $\lambda_{3_{MLCT}}^{em}$ = 786 nm with $\Phi_{3_{MLCT}}^{em}$ = 4.0 × 10^−5^ at room temperature [44]. Emission from this Ru(II),Rh(III),Ru(II) supramolecule is quenched 94 % relative to the model [(Me_2_phen)_2_Ru(dpp)Ru(Me_2_phen)_2_]^4+^ system ( $\lambda_{3_{MLCT}}^{em}$ = 764 nm and $\Phi_{3_{MLCT}}^{em}$ = 7.4 × 10^−4^). The excited state lifetime displays a concurrent reduction for [{(Me_2_phen)_2_Ru(dpp)}_2_RhBr_2_]^5+^ compared to [(Me_2_phen)_2_Ru(dpp)Ru(Me_2_phen)_2_]^4+^ (τ = 22 ns *vs.* 126 ns, respectively) to give *k_et _*= 3.8 × 10^7^ s^−1^.

TL variation alters the observed excited state properties of [{(TL)_2_Ru(dpp)}_2_RhCl_2_]^5+^ and [(TL)_2_Ru(dpp)Ru(TL)_2_]^4+^ although the emissive excited state for these complexes is formally Ru(dπ)→dpp(π*) ^3^MLCT in nature (TL = bpy, phen, Ph_2_phen, or Me_2_phen). The formally Ru(dπ) HOMO donor orbital in the emissive ^3^MLCT excited state contains contributions from the TL π symmetry orbitals which likely produces the observed effects. The Ru(II),Rh(III),Ru(II) structural motif provides intramolecular electron transfer to populate ^3^MMCT excited states at room temperature which is impeded at 77 K. The rate of electron transfer in these systems and the prior dpp and tpp bridged Ru(II),Rh(III) bimetallics are all ca. 10^7^ s^−1^. This indicates the pyrazine portion of the bridge dictates intramolecular electron transfer in this Ru(II),Rh(III) structural motif.

Varying the halide ligand attached to the Rh(III) metal center modulates the energy of the Rh(dσ*) LUMOs and ^3^MMCT energies in this Ru(II),Rh(III),Ru(II) structural motif. Substituting Cl^−^ for Br^−^ decreases the energy of the Rh(dσ*) orbitals as Br^−^ is a weaker σ-donating ligand [26,29,44]. The room temperature steady-state emission spectra of the [{(TL)_2_Ru(dpp)}_2_RhCl_2_]^5+^ and [{(TL)_2_Ru(dpp)}_2_RhBr_2_]^5+^ (where TL = bpy, phen, or Ph_2_phen) show all of these systems display similar energy Ru(dπ)→dpp(π*) ^3^MLCT emissions. The emission intensity of the Br^−^ systems is decreased compared with the Cl^−^ supramolecules. Time-resolved emission spectroscopy displays a decrease in the excited state lifetime of the emission from the ^3^MLCT excited state of the Br^−^ *vs.* Cl^−^ systems at room temperature. This decrease in $\Phi^{em}$ and τ for the Br^−^ trimetallics suggests that the rate of intramolecular electron transfer to populate the ^3^MMCT excited state is modulated by the choice of halide ligand providing for faster electron transfer with a more stabilized ^3^MMCT state and a higher driving force for electron transfer. With the assumption that *k_r_* and *k_nr_* are the same for the [{(TL)_2_Ru(dpp)}_2_RhX_2_]^5+^ trimetallics and the respective [(TL)_2_Ru(dpp)Ru(TL)_2_]^4+^ model, *k_et_* has been calculated with larger values for the Br^−^ *vs.* Cl^−^ systems. With TL = bpy, this variation is from 1.9 × 10^7^ s^−^^1^ for the Cl^−^ and 2.3 × 10^7^ s^−^^1^ for the Br^−^ systems. The 77 K steady-state and time-resolved emission spectroscopy of the [{(TL)_2_Ru(dpp)}_2_RhCl_2_]^5+^ and [{(TL)_2_Ru(dpp)}_2_RhBr_2_]^5+^ trimetallic complexes display emissive properties similar to the respective [(TL)_2_Ru(dpp)Ru(TL)_2_]^4+^ model in a 4:1 EtOH/MeOH rigid matrix as expected if room temperature emission quenching is a result of intramolecular electron transfer.

## 4. Conclusions

The emissive properties of Ru(II) polyazine chromophores provide a useful handle to known excited state electron transfer reactions. The charge transfer nature of these ^3^MLCT excited states can be exploited to promote electron transfer to coupled electron acceptors such as the reported Rh(III) metal centers. Photoinduced intramolecular electron transfer within Ru(II),Rh(III) and Ru(II),Rh(III),Ru(II) complexes has been investigated using room temperature and 77 K steady-state and time-resolved emission spectroscopy. Room temperature emission spectroscopic studies display significant quenching of the emissive ^3^MLCT excited states with respect to the corresponding model systems, allowing determination of rates of intramolecular electron transfer. This requires that the rate of intramolecular electron transfer is competitive with the rate of the radiative and nonradiative decay pathways of these emissive ^3^MLCT excited states. Careful selection of model systems is essential to these studies as the assumption that k_r_ and k_nr_ are the same in the model Ru(II) systems and supramolecular Ru(II),Rh(III) systems is inherent to this analysis. Sample purity is also important in these studies as the rate of electron transfer could be underestimated by the presence of other emissive impurities or overestimated by impurities which quench the emissive ^3^MLCT excited state. Although quenching of the ^3^MLCT emission is observed at RT in fluid solution as a result of intramolecular electron transfer in these Ru(II),Rh(III) supramolecules, at 77 K in a rigid matrix the emissive properties of the Ru(II),Rh(III) and Ru(II),Rh(III),Ru(II) complexes strongly resemble that of their model systems. These observations and the related study of the orbital energetics of these systems suggest intramolecular electron transfer from the emissive ^3^MLCT excited states to populate ^3^MMCT excited states, which occurs at RT in fluid solution and is impeded at 77 K in a rigid matrix.

Emission quenching of several Ru(II),Rh(III) bimetallic complexes as a result of intramolecular electron transfer was observed in a variety of laboratories with somewhat varying conditions. The degree of electronic communication between the Ru(II) and Rh(III) is modulated by the choice of BL. The rate of intramolecular electron transfer depends strongly on the distance between molecular components (r_DA_) within a closely related series of complexes. In the methylene-linked complexes [(bpy)_2_Ru(Mebpy-CH_2_CH(OH)CH_2_-Mebpy)Rh(TL)_2_]^5+^ (TL = bpy, phen) and [(Me_2_phen)_2_Ru(Mebpy-CH_2_CH_2_-Mebpy)Rh(Me_2_bpy)_2_]^5+^, a large difference was observed in *k_et_* with the latter Ru(II),Rh(III) bimetallic displaying *k_et_* an order of magnitude larger than the former. This is attributed by the authors to an increase in the donor-acceptor distance in the systems linked by a three carbon spacer, however the nature of the spacer is also varied in this study. The model used in these systems was a Ru(II) monometallic synthon which provides a good match for the energy and nature of the emissive ^3^MLCT excited state. The addition of a second metal on the remote site of the BL to produce the Ru(II),Rh(III) supramolecules may modulate the rate of nonradiative decay in these systems or change somewhat the nature of the BL acceptor orbital for the emissive ^3^MLCT excited state.

The modification of the linkage in these series of Ru(II),Rh(III) bimetallics likely plays some a in the quenching of the emission in these systems. Phenylene-linked Ru(II),Rh(III) complexes also displayed a strong dependence of the emission quenching on the distance between the two molecular components. As the number of phenylene spacers increased, the rate of intramolecular electron transfer decreased exponentially. These systems provide a series of molecules in which the nature of the linker remains the same and the distance between the donor and acceptor varies. In the phenylene-bridged systems, some contribution to the rapid rate of intramolecular electron transfer may come from the delocalization of the promoted electron in the ^3^MLCT excited state onto the phenylene linker. When comparing the methylene-linked system [(Me_2_phen)_2_Ru(Mebpy-CH_2_CH_2_-Mebpy)Rh(Me_2_bpy)_2_]^5+^ with the analogous phenylene-linked systems [(Me_2_phen)_2_Ru-bpy-(ph)_n_-bpy-Rh(Me_2_bpy)_2_]^5+^ and [(ttpy)Ru-tpy-(ph)-tpy-Rh(ttpy)]^5+^, the intrinsic properties of the BL had a greater influence on *k_et_* than r_DA_. While r_DA_ in the phenylene-linked Ru(II),Rh(III) complexes was larger (15.5 Ǻ) compared to the methylene-linked complex (13.5 Ǻ), *k_et_* was larger in the phenylene-linked systems. This change in the distance dependence with variation of the nature of the linker between the donor and acceptor is somewhat expected given the role of the BL in the optically populated ^3^MLCT excited state and in mediating electron transfer in these systems.

Bridging the ED and EA molecular components using pyrazine-containing BLs displayed stronger electronic communications between the two components. Perturbations to the electrochemical and spectroscopic properties of these Ru(II),Rh(III) systems were indicative of electronic coupling of Ru(II) and Rh(III) metal centers and modulation of the properties of the bridge upon complexation to two metal centers. These systems couple the Rh(III) acceptor directly to the BL involved in the emissive ^3^MLCT excited state. Here the perturbations of the emission energy and orbital energetics within the Ru(II),Rh(III) supramolecular systems provides that the most appropriate model for k_r_ and k_nr_ are the analogous Ru(II),Ru(II) bimetallic systems. The energy and nature of the emissive ^3^MLCT excited state were quite similar for these model systems and the related Ru(II),Rh(III) supramolecular assemblies. It is interesting to note that the bimetallic systems with directly coupled Ru and Rh centers such as [(bpy)_2_Ru(dpp)Rh(bpy)_2_]^5+^, [(tpy)Ru(tpp)RhCl_3_]^2+^ and [(bpy)_2_Ru(dpp)RhCl_2_(phen)]^3+^ all display slower rates of intramolecular electron transfer than might be predicted based on the short donor-acceptor distance in these systems with k_et_ being 2-4 × 10^7^ s^-1^ in all systems despite their varied structures. This may result from the rigid structure of the Rh(III) acceptor with respect to the BL engaged in the emissive ^3^MLCT excited state which may provide for inhibited orbital overlap of the formally π symmetry donor and Rh(dσ*) acceptor orbitals in these systems. These systems also all display stabilized emissive ^3^MLCT excited states with lower driving forces for intermolecular electron transfer to the Rh acceptor which is expected to reduce *k_et_*.

A series of Ru(II),Rh(III),Ru(II) complexes connected through dpp BLs have been studied and emission quenching is observed in these systems. The dpp bridged systems again directly couple the Rh(III) acceptor to the dpp BL engaged in the emissive ^3^MLCT excited state. These trimetallics display significant electronic communication between the Ru(II) and Rh(III) molecular components and modulated BL properties upon complexation to two metal centers. The Ru(II),Ru(II) bimetallics serve as models for the interpretation of the emissive properties of these supramolecules providing similar excited state energies and nature of the emissive ^3^MLCT excited state as the Ru(II),Rh(III),Ru(II) supramolecular assemblies. These Ru(II),Rh(III),Ru(II) supramolecules are shown to function as PECs, collecting reducing equivalents on the Rh center and have been applied to the photoreduction of water to produce hydrogen fuel. These Ru(II),Rh(III),Ru(II) supramolecules undergo efficient intramolecular electron transfer quenching of the emissive ^3^MLCT excited states to populate the ^3^MMCT excited states. In the model Ru(II),Ru(II) bimetallics, the nature of the terminal ligand bound to the Ru impacts the photophysics despite the formally Ru→dpp CT nature of the emissive state in this forum. This likely results from the TL contribution to the formally Ru(dπ), but actually π bonding orbital that serves as the donor orbitals in the emissive ^3^MLCT excited state. This illustrates the care that must be taken in selecting model systems for emission quenching studies. Within the supramolecular Ru(II),Rh(III),Ru(II) systems, the rate of intramolecular electron transfer remains on the order of 1-4 × 10^7^ s^-1^ in this entire series. This indicates that the dpp bridge has a large impact on k_et_ in these systems, somewhat independent of the nature of the other structural components. The choice of the halide attached to the Rh(III) center also influences the rate of intramolecular electron transfer. The use of bromide in place of chloride bound to the Rh center provides for stabilized Rh(dσ*) orbitals and ^3^MMCT excited states with a larger driving force for intramolecular electron transfer. An increase in k_et_ is seen for all series when bromide is substituted for chloride bound to the Rh(III) center.

Steady-state and time-resolved emission spectroscopy provide a probe into the excited state dynamics of Ru(II),Rh(III) and Ru(II),Rh(III),Ru(II) supramolecules. The charge transfer nature of the Ru(II) chromophores lowest lying excited states and the emissive properties of these states provide for a convenient probe of intramolecular electron transfer and a means to direct charge through optical excitation to the site of attachment of an electron accepting Rh(III) center. These ED-BL-EA and ED-BL-EC-BL-ED structural motifs allow efficient photoinduced intramolecular electron transfer that can be monitored through emission spectroscopy. The range of systems studied to date is somewhat limited and more systematic studies of these structural motifs will elucidate more clearly the role that each sub-unit plays in the rate and efficiency of intramolecular electron transfer in these supramolecules. Careful choice of the model systems is essential to the successful application of this emission probe to study intramolecular electron transfer along with care in the assay of the purity of these systems. Study of the RT and 77 K emission properties is very useful to provide additional evidence that the emission quenching observed at RT in fluid solution is a result of electron transfer that will be prohibited at 77 K in rigid media. These Ru(II),Rh(III) assemblies are useful as molecular machines in the design of supramolecular complexes that undergo photoinduced processes to perform complex functions. The intramolecular electron transfer provides for charge separation within the Ru(II),Rh(III) systems allowing them to function as molecular photovoltaics. The coupling of two Ru(II) chromophores to one Rh(III) acceptor in the Ru(II),Rh(III),Ru(II) supramolecules provides for systems that use light to collect reducing equivalents (PEC) and catalyze the multi-electron reduction of water to produce hydrogen.

## Glossary of Abbreviations

bpy: 2,2-bipyridine
BL: bridging ligand
CH_3_CN: acetonitrile
CT: charge transfer
dpp: 2,3-bis(2-pyridyl)pyrazine
E^0-0^: energy gap between ground state and excited state
EA: electron acceptor
EC: electron collector
ED: electron donor
en: energy transfer
et: electron transfer
EtOH: ethanol
GS: ground state
h: Planck’s constant
hν: energy
H_DA_: electron donor-electron acceptor electronic coupling matrix element
HOMO: highest occupied molecular orbital
isc: intersystem crossing
IL: intraligand
k_x_: rate constant for process “x”
LA: light absorber
LF: ligand field
LUMO: lowest unoccupied molecular orbital
Me_2_bpy: 4,4’-dimethyl-2,2’-bipyridine
Me_2_phen: 4,7-dimethyl-1,10-phenanthroline
MeOH: methanol
MLCT: metal-to-ligand charge transfer
MMCT: metal-to-metal charge transfer
nr: nonradiative decay
PEC: photoinitiated electron collector
Ph_2_phen: 4,7-diphenyl-1,10-phenanthroline
phen: 1,10-phenanthroline
PMD: photochemical molecular device
r: radiative decay
r_DA_: donor-acceptor distance
RT: room temperature
rxn: photochemical reaction
T: temperature
TL: terminal ligand
tpp: 2,3,5,6-tetrakis(2-pyridyl)pyrazine
tpy: 2,2’,6’,2”-terpyridine
ttpy: 4'-(*p*-tolyl)-2,2':6',2''-terpyridine
X: halide
ΔG°: Gibbs free energy (thermodynamic driving force)
β: attenuation factor
τ: excited state lifetime
ν_N_: average nuclear frequency factor
κ: electronic transmission coefficient
λ: total reorganization energy
Φ^em^: quantum yield of emission
λ^ab^: absorption maximum
λ^em^: emission maximum

## Appendix

**Appendix 1:** Photophysical properties for Ru(II),Rh(III) and Ru(II),Rh(III),Ru(II) complexes, along with relevant model systems, at room temperature and 77 K.

	RT *^a^*					77 K *^b^*		Ref.
Complex	$\lambda^{abs}$(nm)	$\lambda^{em}$(nm)	$\Phi^{em}$	τ (ns)	k_et_ (s^−1^) *^ c^*	$\lambda^{em}$ (nm)	τ (μs)	
[Ru(bpy)_3_]^2+^	452	605		860				[8]
								
[Ru(bpy)_2_(Me_2_bpy)]^2+ *d*^	456	610		527				[34]
[Rh(bpy)_2_(Me_2_bpy)]^3+ *d*^	318							[34]
[(bpy)_2_Ru(Mebpy-CH_2_CH(OH)CH_2_-Mebpy)Rh(bpy)_2_]^5+ *d*^	456	610		59.9	1.4 × 10^7^			[34]
[(bpy)_2_Ru(Mebpy-CH_2_CH(OH)CH_2_-Mebpy)Rh(phen)_2_]^5+ *d*^	456	610		71.3	1.1 × 10^7^			[34]
[Ru(Me_2_phen)_2_(Mebpy-CH_2_-CH_2_-Mebpy)]^2+^	450	610	0.11	1800		575	7	[35]
[Rh(Me_2_bpy)_2_(Mebpy-CH_2_-CH_2_-Mebpy)]^3+^	298	390				448	2400	[35]
[(Me_2_phen)_2_Ru-(Mebpy-CH_2_-CH_2_-Mebpy) -Rh(Me_2_bpy)_2_]^5+^	450	610	0.00076	6	1.7 × 10^8^	575	6.8	[35]
[Ru(ttpy)_2_]^2+^	490	650	10^−^^6^-10^−^^5^	0.860		629	13.5	[37,38]
[Rh(ttpy)_2_]^3+^	360					521	2.5	[37,38]
[(ttpy)Ru(tpy-tpy)Ru(ttpy)]^4+^	520			600		674	12.9	[37,38]
[(ttpy)Ru(tpy-tpy)Rh(ttpy)]^5+^	520		0.00005	17		674	12.5	[37,38]
[(ttpy)Ru(tpy-(ph)-tpy)Rh(ttpy)]^5+^	495	660	10^−^^6^-10^−^^5^	0.240	≥ 3 × 10^9^	636	13.0	[37,38]
[(ttpy)Ru(tpy-(ph)_2_-tpy)Rh(ttpy)]^5+^	490	650	10^−^^6^-10^−^^5^	1.9	< 5 × 10^8^	629	13.2	[37,38]
[Ru(Me_2_phen)_2_(bpy-ph-bpy)]^2+^	460	642		1.5		600	7	[36]
[Ru(Me_2_phen)_2_(bpy-ph_2_-bpy)]^2+^	460	642		1.5		600	6	[36]
[Ru(Me_2_phen)_2_(bpy-ph_3_-bpy)]^2+^	460	652		1.6		610	7	[36]
[Rh(Me_2_bpy)_2_(bpy-ph-bpy)]^3+^	350					494	40000	[36]
[Rh(Me_2_bpy)_2_(bpy-ph_2_-bpy)]^3+^	350					520	42000	[36]
[(Me_2_phen)_2_Ru(bpy-ph-bpy)Rh(Me_2_bpy)_2_]^5+^	460	640		0.360	3.0 × 10^9^	640	6.9	[36]
[(Me_2_phen)_2_Ru(bpy-ph_2_-bpy)Rh(Me_2_bpy)_2_]^5+^	460	644		2.3	4.3 × 10^8^	644	6.8	[36]
[(Me_2_phen)_2_Ru(bpy-ph_3_-bpy)Rh(Me_2_bpy)_2_]^5+^	460	654		94	1.0 × 10^7^	652	6.8	[36]
[(bpy)_2_RhCl_2_]^+^	326			90		704	19.6	[40]
[(bpy)_2_Rh(dpp)]^3+^	321	450		390		720	24	[40]
[(bpy)_2_Ru(dpp)]^2+^	468	682		382		624	5.36	[40]
[(bpy)_2_Ru(dpp)Ru(bpy)_2_]^4+^	526	790		140		702	2.38	[40]
[(bpy)_2_Ru(dpp)Rh(bpy)_2_]^5+^	514	778		37	2.83 × 10^7^	689	1.71	[40]
[(tpy)Ru(tpp)Ru(tpp)]^4+^	548	830	0.0011	100				[41]
[(tpy)Ru(tpp)RhCl_3_]^2+^	516	830	0.00020	22	4 × 10^7^			[41]
[(bpy)_2_Ru(dpp)Ru(bpy)_2_]^4+^	526	758	0.0014	124		696	2.4	[42]
[(bpy)_2_Ru(dpp)RhCl_2_(phen)]^3+^	509	786	0.00023	30	2.5 × 10^7^	716	1.8	[42]
[(bpy)_2_Ru(dpp)Ru(bpy)_2_]^4+ ^	526	752	0.00098	140		716	2.5	[29]
[(phen)_2_Ru(dpp)Ru(phen)_2_]^4+^	524	750	0.00160	170		695	2.0	[29]
[(Ph_2_phen)_2_Ru(dpp)Ru(Ph_2_phen)_2_]^4+ ^	540	754	0.00173	192		698	2.0	[44]
[(Me_2_phen)_2_Ru(dpp)Ru(Me_2_phen)_2_]^4+ ^	536	764	0.00074	126		710	1.7	[44]
[{(bpy)_2_Ru(dpp)}_2_RhCl_2_]^5+ ^	520	776	0.00026	38	1.9 × 10^7^	730	1.9	[29,42]
[{(bpy)_2_Ru(dpp)}_2_RhBr_2_]^5+ ^	520	776	0.00014	34	2.3 × 10^7^			[29]
[{(phen)_2_Ru(dpp)}_2_RhCl_2_]^5+ ^	520	760	0.00022	35	2.3 × 10^7^	706	1.8	[29]
[{(phen)_2_Ru(dpp)}_2_RhBr_2_]^5+ ^	520	760	0.00017	30	2.8 × 10^7^	706	1.9	[29]
[{(Ph_2_phen)_2_Ru(dpp)}_2_RhCl_2_]^5+^	520	770	0.00024	52	1.4 × 10^7^	696	1.8	[44]
[{(Ph_2_phen)_2_Ru(dpp)}_2_RhBr_2_]^5+ ^	520	770	0.00020	40	2.0 × 10^7^	696	1.9	[44]
[{(Me_2_phen)_2_Ru(dpp)}_2_RhBr_2_]^5+ ^	522	786	0.00004	22	3.8 × 10^7^	724	1.2	[44]

^a^ Measured in acetonitrile at room temperature, unless otherwise noted. ^b^ Measured in 4:1 EtOH/MeOH rigid matrix at 77 K. ^c^ Rate of intramolecular electron transfer calculated using equation 15. ^d^ Measured in water at room temperature.
